# Clinical and Survival Impact of FDG PET in Patients with Suspicion of Recurrent Ovarian Cancer: A 6-Year Follow-Up

**DOI:** 10.3389/fmed.2015.00046

**Published:** 2015-07-22

**Authors:** Daniela Rusu, Thomas Carlier, Mathilde Colombié, Dorothée Goulon, Vincent Fleury, Nicolas Rousseau, Dominique Berton-Rigaud, Isabelle Jaffre, Françoise Kraeber-Bodéré, Loic Campion, Caroline Rousseau

**Affiliations:** ^1^Nuclear Medicine Unit, ICO Cancer Center, Saint-Herblain, France; ^2^CNRS UMR 6299, Centre Régional de Recherche en Cancérologie Nantes-Angers (CRCNA), INSERM U892, Nantes, France; ^3^Nuclear Medicine, University Hospital Nantes, Nantes, France; ^4^Oncology Unit, ICO Cancer Center, Saint-Herblain, France; ^5^Oncologic Surgery Unit, ICO Cancer Center, Saint-Herblain, France; ^6^Statistics Unit, ICO Cancer Center, Saint-Herblain, France

**Keywords:** ovarian cancer, recurrence, PET/CT, 18F-FDG, survival outcome

## Abstract

**Background:**

The aim of this retrospective study was to evaluate the contribution of fluorine-18-fluoro-deoxyglucose (FDG) positron emission tomography (PET) to the clinical management and survival outcome of patients (pts) suspected of recurrent ovarian carcinoma, with the hypothesis that early diagnosis of recurrent ovarian cancer may improve overall survival (OS).

**Methods:**

Fifty-three FDG PET/CT scans were retrospectively analyzed for 42 pts. CT and PET/CT findings were confirmed by imaging and clinical follow-up, and/or pathology, which were considered as the gold standard diagnosis. The treatment plan based on CT staging was compared with that based on PET/CT findings. Medical records were reviewed for pts characteristics, progression-free survival (PFS), and OS. PFS and OS were analyzed using the Cox proportional hazards regression model.

**Results:**

The final diagnosis of recurrence was established pathologically (*n* = 16), or by a median clinical follow-up of 6.5 years (range 0.5-7.5) after the PET/CT (*n* = 37). PET/CT provided a higher detection sensitivity (92.2%, 47/51) than CT (60.8%, 31/51) (*p* < 0.001). Globally, PET/CT modified the treatment plan in 56.6% (30/53) and in 65.2% (15/23) when the CT was negative prior to PET/CT. In 30 cases, those benefited from a modified treatment plan, these changes led to the intensification of a previous treatment procedure in 83.3% (25/30), and to a reduction in the previous treatment procedure in 16.6% of cases (5/30). The Cox regression multivariate analysis showed that the number of lesions visualized by CT and presence of lung lesions detected by PET/CT were significantly associated with PFS (*p* = 0.002 and *p* = 0.035, respectively).

**Conclusion:**

On account of its impact on treatment planning, and especially in predicting patient outcome, FDG PET is a valuable diagnostic tool for cases of suspected ovarian cancer recurrence.

## Introduction

Ovarian cancer has the highest mortality of all gynecological cancers (1). Ovarian carcinoma is usually diagnosed at a late stage due to the paucity and insidious onset of symptoms (2). Despite high-response rates after initial treatment, 20–30% of patients with early-stage disease (stage IA-IIA), and up to 75% of patients with advanced disease (IIB-IV) present with recurrence within 2 years (3). Follow-up protocols usually consist of a physical examination, evaluation of the serum tumor marker (CA125), and morphological imaging techniques, such as ultrasound (US), computed tomography, and/or magnetic resonance imaging (MRI). However, these methods suffer certain limitations. Physical examination may be imprecise, and elevated values of CA 125 are sensitive for the early detection of active disease but do not localize the site of recurrence (4). In addition, conventional imaging (CI) techniques based on anatomical modifications, such as the identification of a new abnormal lesion or changes in the size of a known lesion, may have limited accuracy in the detection of tumor recurrence. Moreover, CT and MRI imaging (either soon after treatment or at later stages) are of limited value for optimally differentiating a recurrence signal from a post-surgical status, or due to their inability to detect normal-sized lymph node metastases (5).

Positron emission tomography (PET) with fluorine-18-fluoro-deoxyglucose (FDG) has been proposed as a way of overcoming these limitations. It has been found to be highly sensitive for detecting recurrent ovarian cancer, especially in patients with an unexplained elevation of serum tumor markers. It offers the combined benefits of anatomical and functional imaging, and has been used to localize areas of increased FDG with improved anatomical specificity, and to exclude disease in sites of residual structural abnormality (6). A recent study evaluated the clinical impact of FDG PET upon treatment strategy, and found that accurate localization of ovarian cancer recurrence impacts both patient outcome and treatment strategy (7). The purpose of this study was to assess the clinical and therapeutic impact of FDG PET in women who were suspected of ovarian cancer recurrence.

## Materials and Methods

### Patient population

Patients included retrospectively were of over 18 years of age, with histologically proven ovarian cancer that was suspected of recurrence, and had been treated in our institution between 2006 and 2010. All patients of the institution received written information on the possibility of use of their medical data in retrospective studies. After initial diagnosis, the patients were monitored by physical examination, CA 125 assay, and CT every 6 months over a 5-year period. Recurrent disease was suspected following abnormal examination results and/or symptoms suggestive of recurrence, equivocal results on CI, and/or the isolated elevated serum tumor marker (CA 125). PET scans were acquired only if recurrence was suspected from the different criteria described above. The International Federation of Gynecology and Obstetrics (FIGO) classification was used for clinical staging (8). A minimum of 6 months follow-up after the post-treatment PET scan was also an inclusion criterion for this study.

### CT imaging

CT images were obtained using a Hi-speed CT scanner (GE Medical Systems, Milwaukee, WI, USA). Patients fasted for ≥4 h before CT imaging. Scans were acquired in the craniocaudal direction, between the diaphragm and the perineum. After a series of unenhanced sections, all patients received intravenous bolus injection of contrast medium (Omnipaque 300™, GE Healthcare, Vélizy-Villacoublay, France) at a rate of 2.5–3 mL/s and a volume of 75–90 mL. The Hi-Speed CT scanner generated contiguous slices measuring 5 mm in thickness and reconstruction interval was 1.25 mm.

### PET/CT scanning

Scanning, from the patient’s head to the pelvic floor with their arm held above their head, was performed using a Siemens Biograph mCT40 fitted with a 20 cm axial field of view, true of flight (TOF) feature, and in-plane resolution of 4.4 mm in full width at half maximum (Siemens, Knoxville, TN, USA). Images were reconstructed with Ultra HD and TOF (3 iterations, 21 subsets). Patients fasted for at least 4 h before PET acquisition. Fasting blood glucose, which had to be <7 mmol/L, was checked prior to injection of 3 MBq/kg of FDG. Intravenous injection was followed by a tracer-uptake period of approximately 60 min, during which patients remained seated in a quiet room. Low dose-unenhanced CT was performed for localization and attenuation correction. The reformatted, transverse, coronal, and sagittal views were used for interpretation.

### Qualitative imaging analysis

CT and PET/CT clinical reports were collected and retrospectively analyzed. All PET/CT images were interpreted independently by two nuclear medicine physicians. The observers were aware of the clinical data and findings obtained with other anatomic imaging modalities. When abnormal FDG uptake was observed, its exact anatomical location was indicated on the CT scans. On PET images, the presence of a relapse tumor was suspected when accumulation of FDG was moderately to markedly increased, in comparison with that of comparable normal contralateral structures or surrounding tissues, excluding physiological bowel and urinary activity.

For both ethical and practical reasons, not every suspected involved lesion was evaluated by histology. A gold standard reference was therefore established based on histology and 6 months follow-up data (significant tumor progression on clinical examination or on CT according to RECIST Criteria). True-positive results corresponded to an abnormal image by an imaging method (CT or PET/CT) confirmed by histopathology or follow-up. For example, foci that were detected by PET/CT, which were not histologically examined, were considered to be true-positives if the disease became obvious upon clinical observation or with the imaging methods during the follow-up. A negative finding on an imaging method was considered to be false-negative if positive by histopathology or follow-up. Indeed, a negative finding on an imaging method that was detected by another imaging method and confirmed by histopathology or by a clinical or imaging progression was considered to be false-negative. True-negative results corresponded to the absence of an abnormality by an imaging method, confirmation by a negative tissue biopsy, or lack of recurrence during the follow-up. For example, when no abnormality was found by PET/CT, or when no intervention was performed, the result was considered to be a true-negative if no disease was identified by other imaging studies or by clinical observation during 6 months follow-up. Finally, an abnormal result on an imaging method was considered to be false-positive if negative by histopathology or by follow-up. Suspicious involved lymph nodes were followed by CT although their size initially was not enough RECIST 1.1 criteria. Indeed, their development after treatment or not treatment has defined their status.

### Statistical analysis

Results are expressed as mean values ±SD or median (range) in the case of small group sizes. Qualitative variables were compared using the Pearson test or the Fisher exact test where necessary (small sample size). Paired qualitative variables were compared using the Mac Nemar test. Quantitative variables were compared using the Student’s *t*-test or the Mann–Whitney *U* test where necessary. Survival curves were calculated by means of the Kaplan–Meier method and compared by a log rank test. Multivariate analysis was performed by means of a Cox regression model. Analyses were performed using SAS 9.1 (SAS Institute Inc., Cary, NC, USA) and Stata/SE 10 (StataCorp LP, College Station, TX, USA). All tests were performed at a two-sided significance level of α = 0.05.

## Results

### Patient characteristics

We retrospectively evaluated 42 patients, with a median age of 45.5 years (35–81). For these patients, 53 PET/CT were performed. Patient characteristics are described in Table 1. All patients, except one, had a high risk of relapse. The recurrence was suspected due to abnormal results by CI in 30/53 PET/CT (56.6%) and an isolated elevated serum tumor marker CA 125 was observed in 23/47 PET/CT (48.9%). Recurrence was histopathologically confirmed in 30% (16/53) of cases, or by clinical follow-up for other patients, with a median clinical follow-up of 6.5 years (range 0.5–7.5) after PET/CT. PET/CT scans were not repeated during the follow-up even in cases of negative PET/CT results. Only CT or MRI was repeated. The median delay between PET/CT and initial treatment was 2.72 years (range 0.2–21.1).

**Table 1:** **Patient characteristics before each FDG PET (53 examinations)**.

Characteristics	Value
Number of patients	42
Number of FDG PET	53
Median years (range)	59.3 (35.1–81)
Tumor histology	
Adenocarcinoma	37
Borderline	3
Serous	1
Granulosa cells	1
FIGO stage	
II	1
III	41
CA 125 level before FDG PET (Normal <1.5 μg/L)	
Positive	47
Negative	6
Time from previous treatment (months)	
Median (range)	33 (2–253)

### Imaging results

Table 2 shows the patient-based performance of whole-body PET/CT and CI. Whole-body PET/CT provided higher sensitivity and accuracy compared to CI (*p* < 0.001), especially with a sensitivity of 92.2% for PET/CT compared to 60.8% for CI. The four PET/CT false-negative results were local recurrences, all of which were also negative by CT.

**Table 2:** **Performance of FDG PET/CT and conventional imaging to detect recurrence of ovarian cancer**.

Performances	PET/CT whole body	Conventional imaging	*p*
*N*	53		
True-positive (*n*)	47	31	
True-negative (*n*)	2	0	
False-positive (*n*)	0	2	
False-negative (*n*)	4	20	
Sensitivity (%)	92.2	60.8	<0.001
Specificity (%)	100	–	0.333
PPV (%)	100	93.9	0.167
NPV (%)	33.3	–	0.046
Accuracy (%)	92.5	52.8	0.01

**Figure 1:**
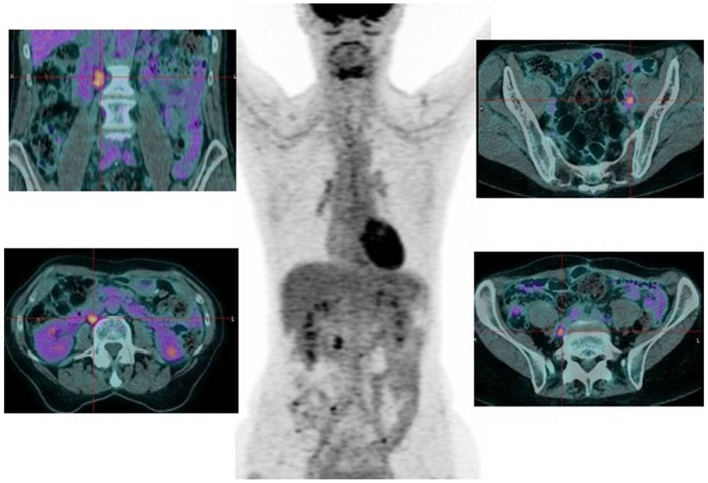
**Seventy-two-year-old patient suspected of pelvic recurrence of ovarian cancer**. The CT was negative and CA125 normal (12 U/mL for *N* < 35), but with increasing kinetic. PET/CT found nodal retroperitoneal and pelvic abnormalities.

For patients with elevated tumor marker CA125 (*n* = 47), the sensitivity of PET/CT was 91.3% (42/47), compared to 50% (23/46) for CI. PET/CT accurately diagnosed a recurrent disease in 91.5% of patients (43/47) compared to 48.9% (23/47) by CI (*p* < 0.001). The most frequent site of recurrence was in the lymph nodes (as described in Table 3; Figure 1).

**Table 3:** **Frequency of abnormal FDG PET findings by the site of involvement**.

Sites of recurrence	Frequency
Lymph nodes	38 (71.7%)
Supradiaphragmatic	5
Supra and infradiaphragmatic	12
Pelvis	7 (13.2%)
Liver	10 (18.9%)
Bone	2 (3.7%)
Lung	4 (7.5%)
Peritoneal lesion	16 (30.2%)

### Clinical impact of PET/CT

Table 4 summarizes the changes in patient management based on PET/CT results. The confirmation of recurrence sites by biopsy was not considered as a modified treatment plan. Therefore, the therapeutic strategy was modified in 30/53 (56.6%) cases, and in 65.2% (15/23) when CI was negative. In 30 cases, those benefited from a modified treatment plan, these changes led to the use of a previously unplanned therapeutic procedure in all cases, to the intensification of a previous treatment procedure in 83.3% (25/30) of cases, and to a reduction in previous treatment procedures in 16.6% of cases (5/30). In true-positive cases, the treatment showed decreases lesions size (for lesion without possible biopsy by ethics), decreases of biomarker with treatment, decreases of not significant nodes size with RECIST 1.1 but positive for PET/CT and sometimes appearance of significant lesions on CT (whereas PET/CT was earlier positive) in case of non-responders to treatment.

**Table 4:** **Changes in patient management based on the PET/CT results**.

Treatment proposed before PET FDG	Therapeutic strategy decided after PET FDG
11 (Surgery)	7 (Chemotherapy)
	1 (Surgery and chemotherapy)
	1 (Extended surgery)
	2 (Surveillance)
3 (Chemotherapy)	2 (Surgery)
	1 (Surveillance)
1 (Radiotherapy)	1 (Chemotherapy)
15 (Surveillance)	13 (Chemotherapy)
	1 (Surgery)
	1 (Surgery and chemotherapy)

### Survival analysis

The impact of several variables on patient survival [progression-free and overall survival (OS)] were analyzed: age, increased CA125, the number of CT lesions, the number of PET/CT lesions, and semi-quantitative indexes, such as SUVmax, SUVmean, SUVpeak, and total lesion glycolysis (TLG).

**Figure 2:**
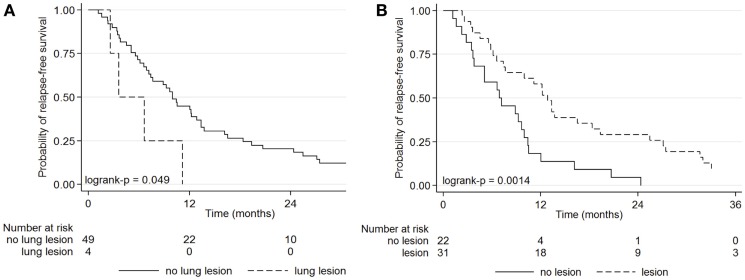
**Kaplan–Meier curves for progression-free survival for patients with a suspected ovarian cancer recurrence according to the presence or absence of lung lesions by FDG PET (A), and the presence or absence of lesions by CT (B)**.

Lung lesions detected by FDG or abnormal CT were predictive of progression free survival (PFS) (Figure 2). The PFS was better not only for patients with PET findings with lung foci (*p* = 0.035) but also for patients with abnormal CT than with normal CT (*p* = 0.002), as described in Table 5. The 1-year PFS was 42.9% for patients with PET findings without lung lesions, and 0% with lung metastases (*p* = 0.049).

**Table 5:** **Uni- and mutivariate analyses for the determination for factors prognostic of progression-free survival (PFS) in recurrent ovarian cancer**.

	Univariate analysis		Multivariate analysis	
Parameters	HR (95% CI)	*p*-Value	HR (95% CI)	*p*-Value
Age	0.99 (0.96–1.02)	0.514	–	–
CA 125 > 35 vs. ≤35	2.47 (0.86–7.09)	0.093	–	–
FDG PET foci				
>0 vs. 0	0.79 (0.33–1.87)	0.590	–	–
Lung lesion >0 vs. 0	2.77 (0.96–7.96)	0.059	3.14 (1.08–9.11)	0.035
CT foci 0 vs. >0	0.39 (0.21–0.71)	0.002	2.68 (1.45–4.96)	0.002
SUVmax	1.01 (0.95–1.06)	0.891	–	–
SUVmean	1.02 (0.94–1.10)	0.680	–	–
SUVpeak	1.01 (0.95–1.08)	0.676	–	–
TLG	1.00 (0.99–1.00)	0.804	–	–

**Figure 3:**
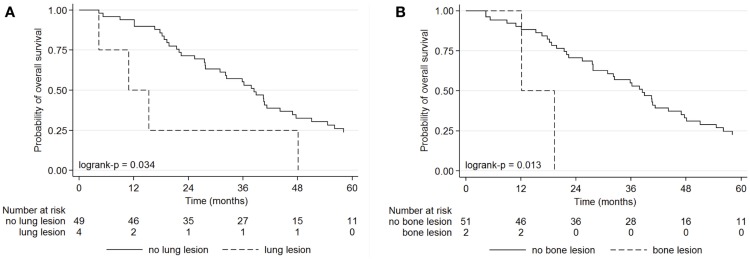
**Kaplan–Meier curves for overall survival of patients with a suspected ovarian cancer recurrence according to the presence or absence of lung lesions on FDG PET (A), and the presence or absence of bone lesions on FDG PET (B)**.

Bone or lung lesions detected only by FDG PET were predictive of OS, whereas CI was not contributive (Figure 3). The OS was better for patients with PET findings without FDG foci corresponding to lung or bone metastasis (unknown by CI) than with PET findings, with lung foci (*p* = 0.034) or with bone foci (*p* = 0.020), as described in Table 6. The 1-year OS was 90.2% in patients with PET findings without bone lesions, and 50.0% with bone metastases.

**Table 6:** **Uni- and mutivariate analyses for the determination of factors prognostic for overall survival (OS) in recurrent ovarian cancer**.

	Univariate analysis		Multivariate analysis	
Parameters	HR (95% CI)	*p*-Value	HR (95% CI)	*p*-Value
Age	1.02 (0.98–1.04)	0.298	–	–
CA 125 > 35 vs. ≤35	1.67 (0.59–4.70)	0.970	–	–
FDG PET foci				
>0 vs. 0	1.15 (1.02–1.29)	0.023	–	–
Lung lesion >0 vs. 0	2.93 (1.03–8.34)	0.044	3.12 (1.09–8.89)	0.034
Bone lesion >0 vs. 0	5.53 (1.22–25.12)	0.027	6.09 (1.33–27.83)	0.020
CT foci >0 vs. 0	0.63 (0.34–1.15)	0.129	–	–
SUVmax	1.02 (0.97–1.07)	0.415	–	–
SUVmean	1.00 (0.92–1.10)	0.904	–	–
SUVpeak	1.03 (0.97–1.09)	0.308	–	–
TLG	1.00 (0.99–1.00)	0.196	–	–

## Discussion

To our knowledge, this study is the first of its kind to extensively follow-up suspected recurrent ovarian carcinoma by PET/CT imaging, with the goal of determining prognostic factors for disease-free and OS, as well as the clinical impact of FDG PET in this indication. There are two major findings in this current study: (1) the absence of lung recurrence sites by PET/CT imaging was independent and prognostic of a good PFS (42% 1-year PFS) and (2) the absence of bone lesions by whole-body FDG PET was an independent and robust prognostic factor (90.2% 1-year OS).

Of all the variables analyzed (age, CA 125, number of target lesions on CT, abnormalities on CT, number of target lesions on PET/CT, abnormalities on PET/CT, lymph node, peritoneal, liver, pelvis, lung, or bone metastases, as well as semi-quantitative indexes, such as SUVmax, SUVmean, SUVpeak, and TLG), lung lesions detected only by PET/CT were significantly associated with PFS, while bone or lung metastases detected only by PET/CT were associated with OS. The semi-quantitative index of PET/CT has no prognostic value in our study. One possible explanation is the heterogeneity of the population studied, with one or more relapses per patient, different types of treatments, and different disease resistance to treatment. The results of our study are consistent with that of Hebel et al. (9). They did not find any significant association between SUVmax and the specific survival in patients with a positive PET/CT scan, nor a significant difference between the SUVmax of the most intense lesion or the number of suspicious lesions on PET/CT scan between patients who responded or not to therapy. On the contrary, the study of Sala et al. showed a possible prognostic role for SUVmax (10). For patients with low-grade serous ovarian cancer, the TLG could be prognostic for survival after relapse, according to a study by Takeuchi (11). In their study, multiple recurrence was associated with poorer PFS than single-lesion recurrence, while CA 125 and SUVmax were not significant predictors of PFS or OS. For our group of patients, TLG was not associated with either disease-free survival or OS (12). In the study by Levy et al., CA 125 level of recurrence and the pattern of CA 125 elevation were significantly associated with PFS and OS by univariate analysis, while in the multivariate analysis increases in CA 125 above normal levels was an independent predictor of PFS and of OS (Levy). In our study, CA 125 had no prognostic value. Sala et al. found that the number, size, and SUVmax of recurrent ovarian cancer lesions in the peritoneum and distant lymph nodes were significantly associated with OS and PFS [Sala 8]. The recent study of Kim et al. found that the platinum-free interval, type of second-line treatment, metabolic tumor volume, and TLG were all significant prognostic factors for post-relapse survival (13).

According to clinical guidelines, when recurrence is suspected, the second step after the determination of CA 125 levels is the CT scan (14–16), with MRI as an alternative if the CT is contraindicated (17). However, several studies have shown the diagnostic superiority of PET/CT over CT. There are some limitations of PET/CT, including the non-specific nature of tracer-uptake, which can accumulate in sites of inflammation and infection, which may be detected as false-positive (18). In addition, PET/CT has a limited ability to detect small lesions, particularly for those <5 mm, with a false-negative rate of 5–10% (19, 20). PET/CT resolution is currently around 4 mm, and even with technical improvements, it cannot drop below 2 mm because the free path of a positron will always leave doubt as to its place of origin (21). Sometimes, the fusion of PET and CT images is not perfect, because of the physiological digestive peristalsis. False-negative results are possible in clear cell and mucinous ovarian cancer, and in necrotic or cystic lesions (22–24). Thus, in a prospective study, Risum et al. evaluated the sensitivity and specificity of US, CT, and PET/CT in the diagnosis of recurrence in 60 patients (25). While the specificity was the same (90%) for the three tests, sensitivity was 97% for PET/CT, 81% for CT, and 66% for US. In addition, PET/CT was superior to single recurrence detection and found recurrence in 50% of patients with negative CT and multifocal recurrence in 42% of cases with isolated recurrence on CT. The meta-analysis of Gu et al., who compared the CA 125, PET, PET/CT, and MRI for the diagnosis of recurrent ovarian cancer in 882 patients found a higher sensitivity for PET/CT (91%) and greater specificity for CA 125 (5). The retrospective study of Antunovic et al. showed better sensitivity (82 vs. 69%), specificity (87 vs. 47%), and accuracy (83 vs. 66%) of PET/CT compared to CI, and better sensitivity compared to CA 125 (83 vs. 59%). Furthermore, the sensitivity of the PET/CT was not affected in this tumor differentiation study (26). PET/CT combined with contrast iodine injection was superior in the detection of recurrence compared to PET/CT without injection in the study by Kitajima et al. (121 patients), with a sensitivity of 87 vs. 78% (27). Contrast iodine injections were not performed in our PET/CT analysis. The diagnostic value of PET/CT in our study (sensitivity 97.5%, 47 patients) was similar to that of MRI in the study of Sanli et al. (28), and even better for the detection of peritoneal carcinomatosis lesions of between 0.5 and 2 cm (*p* < 0.05). PET/CT has good sensitivity in detecting lymph node metastases (29), especially supra-diaphragmatic lesions (30). In our study, in 71.7% of cases, there were lymph nodes metastases, with 32% of cases with supra-diaphragmatic lesions. A negative FDG PET/CT has a high-negative predictive value (9). The results of our study are comparable to the literature, with a good performance of PET/CT (Table 7) (5, 6, 9, 11, 23, 25, 26, 28, 31, 32). Our study found a significant impact of PET/CT in the management of recurrent ovarian cancer (Table 8). The therapeutic procedures were changed in 30/53 of our cases (56.6%), which is comparable with the previously published studies of Bilici – 51.6%, Ebina – 58.4%, Fulham – 58.9%, Hebel – 69%, and Rousseau – 71.5% (6, 7, 9, 33, 34).

**Table 7:** **Sensitivity of PET/CT in the detection of recurrent ovarian cancer: comparison with previously published data**.

Studies	No. pts	Sensitivity PET/CT (%)
Gu et al. (5)	882	91
Gouhar et al. (23)	39	90
Rubini et al. (31)	79	85
Hebel et al. (9)	48	97
Takeuki et al. (11)	48	94
Risum et al. (25)	60	97
Antunovic et al. (26)	121	82
Sanli et al. (28)	47	97.5
Sari et al. (32)	34	96.1
Bilici et al. (6)	60	95
Our study	42	92.2

**Table 8:** **Impact of PET/CT in the management of recurrent ovarian cancer: comparison with previously published data**.

Studies	No. pts	Change of treatment (%)
Fulham et al. (7)	90	58.9
Hebel et al. (9)	48	69
Bilici et al. (6)	60	51.6
Rousseau et al. (33)	34	71.5
Ebina et al. (34)	44	58.4
Our study	42	56.6

Despite its high accuracy (80%) in the diagnosis of ovarian cancer recurrence (35), CA 125 has its limits. While a normal value cannot exclude the presence of disease, an increase of CA 125, even in normal limits, can predict recurrence. According to a study of Bhosale et al., PET/CT detected recurrence in 58% of cases when CA125 was normal, while 31% of patients with normal CA 125 and negative CT had ovarian cancer recurrence confirmed by histology (36). Furthermore, high values cannot differentiate local recurrence from distant metastases (14, 31). It is the role of imagery, and especially PET/CT, to answer that question.

Our study has several limitations. First, it is a retrospective study with potential inherent biases, and further prospective studies are needed to confirm the results. Second, the ideal gold standard for any analysis is histological confirmation (*n* = 16/53). However, clinical follow-up (*n* = 37/53) is also a valid way to evaluate diagnostic accuracy and response to therapy, but it would have been unethical to investigate all PET/CT detected foci using invasive procedures. Finally, the number of patients in the study was relatively small and it warranted the necessity to gather patients for survival analysis in order to retain a valid and strong power by statistical analysis.

## Conclusion

In recurrent ovarian carcinoma suspected by elevated CA 125 serum levels and normal or dubious CT, FDG PET/CT provides staging information that more accurately stratifies prognostic risk in recurrence ovarian cancer when compared with CT alone. There is also a strong evidence to support the use of PET/CT not only for the detection of recurrent ovarian cancer but also to confirm recurrence from the point of view of the therapeutic outcome (choice of treatment). Nonetheless, further evaluations appear necessary.

## Conflict of Interest Statement

The authors declare that the research was conducted in the absence of any commercial or financial relationships that could be construed as a potential conflict of interest.
